# Reboot coaching programme: a mixed-methods evaluation assessing resilience, confidence, burnout and depression in medical students

**DOI:** 10.1177/00369330231213981

**Published:** 2023-12-05

**Authors:** Judith Johnson, Lucy Pointon, Rebecca Talbot, Rebecca Coleman, Luke Budworth, Ruth Simms-Ellis, Katharina Vogt, Dialechti Tsimpida, Chandra Shekha Biyani, Reema Harrison, Gloria Cheung, Colin Melville, Vijay Jayagopal, William Lea

**Affiliations:** 1 School of Psychology, 4468 University of Leeds, Leeds, UK; 2 Yorkshire Quality and Safety Research Group, 170791 Bradford Institute for Health Research, Bradford Royal Infirmary, Bradford, UK; 3 School of Public Health and Community Medicine, University of New South Wales, Sydney, Australia; 4 School of Justice, Security and Sustainability, 7703 Staffordshire University, Stoke-on-Trent, Staffordshire, UK; 5 School of Healthcare, 4468 University of Leeds, Leeds, UK; 6 1343 Pennine Care NHS Foundation Trust, Humphrey House, Bury, UK; 7 Department of Public Health, Policy and Systems, University of Liverpool, Liverpool, UK; 8 Department of Urology, St James's University Hospital, 4472 Leeds Teaching Hospitals NHS Trust, Leeds, UK; 9 Centre for Health Systems and Safety Research, Australian Institute of Health Innovation, Faculty of Medicine, Health and Human Sciences, 7788 Macquarie University, NSW, Sydney, Australia; 10 12195 Hull York Medical School, University Road, Heslington, York, UK; 11 Faculty of Biology, Medicine and Health, 5292 Manchester University, Manchester, UK; 12 York Hospital, 8749 York and Scarborough Teaching Hospitals NHS Foundation Trust, Clifton, York, UK

**Keywords:** Medical education, medical students, burnout, depression, resilience

## Abstract

**Background:**

Poor mental health in medical students is a global concern. Effective interventions are required, which are tailored towards the training-related stressors medical students experience. The Reboot coaching programme is an online, tailored intervention based on cognitive-behavioural principles.

**Aims:**

To evaluate whether the Reboot coaching programme tailored for medical students was feasible and associated with improvements in mental health outcome indicators.

**Methods:**

Medical students participated in two group online workshops and a one-to-one coaching call with a Reboot-trained licensed psychological therapist. Participants provided data at: baseline (T1), post-workshops (T2), post-coaching call (T3) and 4-month follow-up (T4). Outcome measures included resilience, confidence, burnout and depression. Feedback was provided regarding the workshops at T2.

**Results:**

115 participants (93/80.9% women; *m*age = 23.9; *SD *= 2.8) were recruited, 83 (72.2%) completed all intervention elements and 82 (71.3%) provided T4 data, surpassing recruitment and retention targets. There were significant improvements following baseline in resilience (*ps *< .001), confidence (*ps *< .001), burnout (*ps *< .001) and depression (*ps *≤ .001). Most participants agreed the workshops imparted useful skills (n = 92; 99%) and would recommend Reboot to others (n = 89; 95.6%).

**Conclusions:**

Existing interventions have produced mixed results regarding their effectiveness in improving medical students’ mental health. Reboot is a feasible intervention in this group which is associated with improvements in resilience, confidence, burnout and depression. Further controlled studies of Reboot are now needed.

Poor mental health in medical students is a significant problem globally.^1^ Burnout affects 50% of medical students,^2^ one-third experience depression and 10% report suicidal ideation.^3,4^ Several factors contribute to poor mental health in this group, including a high academic workload and a tendency towards maladaptive perfectionism.^5,6^

Stressful clinical events on placement are also a key risk factor for poor mental health in medical students.^5^ These events are those which are intrinsic to medical work, including experiencing death and working with dying patients^7,8^; involvement in medical errors^9^ and unprofessional behaviour from colleagues.^7^ However, there are no available evidence-based interventions to support medical students with placement-related stressors; the mental health interventions which have been tested in this group have been largely generic and ‘off-the-shelf’, including mindfulness, relaxation training and yoga.^10^ These have been criticised for lacking relevance and failing to address those stressors intrinsic to medical training and practice.^11,12^ As such, there is an urgent need for tailored interventions which help students prepare and support them with the specific occupational stressors they will encounter during their placements, training and beyond.

To address this gap, we adapted and evaluated the feasibility of implementing the Reboot (**Re**covery-**BOO**sting **T**raining) Coaching Programme for medical students, to enhance their preparedness for involvement in and recovery after stressful healthcare events. Reboot combines two online group workshops and a one-to-one follow-up coaching phone call, maximising the benefits of peer engagement in the group workshop and the opportunity for professional, confidential support in the call. In contrast to existing psychological interventions for medical students, Reboot has been designed specifically for healthcare professionals and students, drawing on a synergy of (1) research into the impacts of stressful healthcare events, (2) psychological resilience theory and (3) evidence-based cognitive-behavioural techniques. Importantly, Reboot addresses the stressful events which are inherent in healthcare professionals’ and students’ work, providing support for those events which cannot be targeted with organisational interventions.

We anticipated Reboot would be beneficial for medical students for four reasons. First, its pro-active, prevention-focused approach reduces stigma in participating; a key factor known to reduce participation in medical student groups.^4,13,14^ Second, it targets the reduction of maladaptive perfectionism, known to contribute to poor mental health in medical students.^6^ Third, it uses materials and cases studies which are specific to the challenges medical students face on placement; it is experienced as engaging and relevant for students in a way which other interventions cannot be.^15,16^ Fourth, it is all delivered online, improving ease of access and opportunities to discuss experiences with medical students from different regions.

The overarching aim of the current study was to evaluate Reboot in medical students. Our *primary* objective was to assess whether it was feasible to deliver Reboot to medical students. Our *secondary* objective was to assess whether participating in Reboot was associated with increased self-reported psychological resilience, confidence in coping with adverse events, and decreased burnout and depression.

## Methods

### Design

A single-arm, before-after feasibility design, including a mixed-methods evaluation guided by the widely used Kirkpatrick model for assessing training interventions.^17^ This recommends evaluating outcomes at four levels (reaction, learning, behaviour, results), and has been used in previous Reboot evaluations.^15,16,18^

### Intervention

Reboot included two, 2-hour online group workshops and a one-to-one coaching call over 4 weeks. Group workshops involved exercises based around tailored case studies, which were adapted for the target sample. In this study, case studies were initially adapted by JJ and WL and then refined via feedback from focus groups (n = 4) with medical students (n = 7), junior doctors (n = 2) and medical educators (n = 3), and from one-to-one semi-structured interviews with medical students (n = 3), junior doctors (n = 2) and one medical educator (conducted by RT). The workshops introduced the intervention, involving individual and group-based exercises which were then personalised in the coaching video/phone call. Participants were allocated to workshop groups of 5–10 participants. For further intervention description, please see Appendix 1.

### Participants, procedure and ethics

Participants were recruited UK-wide via medical schools and were from years involving clinical placements. Medical schools were contacted by email and via email cascade from the UK Medical Schools Council (MSC). We collected online questionnaire data at four-time points: Baseline (T1), on completion of the two group workshops (T2), on completion of an individual coaching call (T3), and 4 months post-baseline (T4). The study was approved by the School of Psychology, University of Leeds Ethics Committee (date: 19/01/2022; approval number: PSYC-542).

### Sample size

We aimed to recruit ≥81 participants, based on a power analysis for a one-group repeated measures ANOVA main effect, assuming a correlation between pre- and post-intervention scores of *r *= 0.5; a power of 1 − *b *= 0.9 and a small effect size of *f *= 0.15. This calculation accounted for potential participant drop-out. Anticipating medium-large effect sizes^15^; with 81 participants we could still detect a medium effect (*f *= 0.25) with a power of 1 − *b *= 0.9 with around 50% dropout at T4.

### Primary feasibility outcomes

Our primary feasibility outcomes included demand (number of expressions of interest), recruitment (number consenting, completing baseline measures and attending the first workshop) and retention (defined as the number: (1) participating in all intervention elements and (2) completing T4 outcome indicators). Consistent with previous evaluations,^15,18^ feasibility was established if the following criteria were met:

-  ≥ 80 expressions of interest
-  ≥ 80 recruited
-  ≥ 70% completing all intervention elements
-  ≥ 50% completing all T4 measures.

### Secondary outcomes

*Resilience*. The six-item Brief Resilience Scale (BRS) measures perceptions of personal resilience, including ‘I tend to bounce back quickly after hard times’. It has good concurrent validity with other resilience questionnaires^19^ detects learning changes^15,16^ and had good internal reliability in the present study (Cronbach's alphas = 0.85–0.87). Resilience was measured at T1, T3 and T4. Items are scored from 1 to 5 and cut-offs for the BRS are based on mean scores:1.00–2.99 = low resilience; 3.00–4.300 = normal resilience and 4.31–5.00 = high resilience.

*Confidence*. The three-item Confidence in Coping with Adverse Events scale (CAE) includes items such as ‘If I was involved in an adverse event for which I thought I held some responsibility I know the things I would do to help manage my stress levels’.^15,16^ It has acceptable internal reliability (Cronbach's alphas = 0.47–0.69) and detects learning changes.^15,16^ Confidence was measured at all time points.

*Burnout.* We included six items from the Oldenburg Burnout Inventory (OLBI), the three highest loading items on each subscale of ‘exhaustion’ and ‘disengagement’. Items included ‘After my work, I usually feel worn out and weary’. The present version has been used in previous evaluations to detect change.^15,16^ It had acceptable internal reliability in the present study (Cronbach's alphas = 0.63–0.74). Burnout was measured at T1, T3 and T4.

*Depression.* The 9-item Patient-Health Questionnaire-9 (PHQ-9) is widely used to measure depression.^20–22^ It captures change^21^ and had good internal reliability in the present study (Cronbach's alphas = 0.84–0.88). Depression was measured at T1, T3 and T4. Total scores of 0–4 suggest no depression, 5–9 suggest mild depression, 10–14 suggest moderate depression, 15–19 suggest moderate-severe depression and >20 suggest severe depression.

*Reactions to the training questionnaire*. This contained eight items exploring how relevant, useful and engaging the training was (see Table 3) which have been used in previous evaluations.^15,16,18,23^ This was administered at T2.

### Analysis plan

Descriptive analyses were calculated and reported. To assess for changes over time, we employed simple random intercepts linear mixed models (restricted maximum likelihood estimation) through the R lme4 package.^24^ Each outcome was modeled separately, and timepoint as a categorical variable was our main predictor (baseline, T1 and post-intervention time-points: T2, T3 and T4). Analyses were conducted both unadjusted for any demographic variables, and subsequently adjusted for key variables (age, gender). Analyses were conducted using participants with complete data (‘CC’) only, or as a conservative sensitivity analysis, carrying participants’ last recorded outcome score forward to subsequent time points (‘LPCF’). Model-implied marginal means were compared for all outcomes in Holm-adjusted post hoc t-tests for all types of models. Estimates of standardised mean differences (Cohen's ds) are outlined and were calculated as the average difference in outcomes between two comparator time points (e.g., CAE mean T1 minus T3), divided by the standard deviation of differences.

## Results

There were 262 expressions of interest, 131 completed baseline questionnaires and 115 (93/80.9% women; *m*age 23.9; *SD *= 2.8) were finally recruited. There was greater demand for Reboot than spaces available. There were 8 (7.0%) participants in year 3 of their degree, 46 (40.0%) in year 4, 54 (47.0%) in year 5. Six participants were in other years (5.2%) and one preferred not to say (0.9%). There were 57 (49.6%) White participants, 27 (23.5%) Asian or Asian British participants, 13 Black or Black British participants (11.3%) and 15 (13.0%) describing their ethnicity as ‘other’. Three (2.6%) preferred not to say. There were 42 (36.5%) participants from Northern England, 25 (21.7%) from Southern England, 35 (30.4%) from the Midlands, 9 (7.8%) from Scotland, 1 (0.9%) from Wales and 3 (2.6%) preferring not to say.

*Primary feasibility outcomes: Demand, recruitment and retention*. Expressions of interest (n = 262) and recruited participants (n = 115), both surpassed the demand and recruitment targets of 80. Of the 115 participants recruited, 83 (72.2%) completed all intervention elements and 82 (71.3%) provided T4 data, surpassing retention targets of 70% and 50%, respectively.

**Figure 1.**
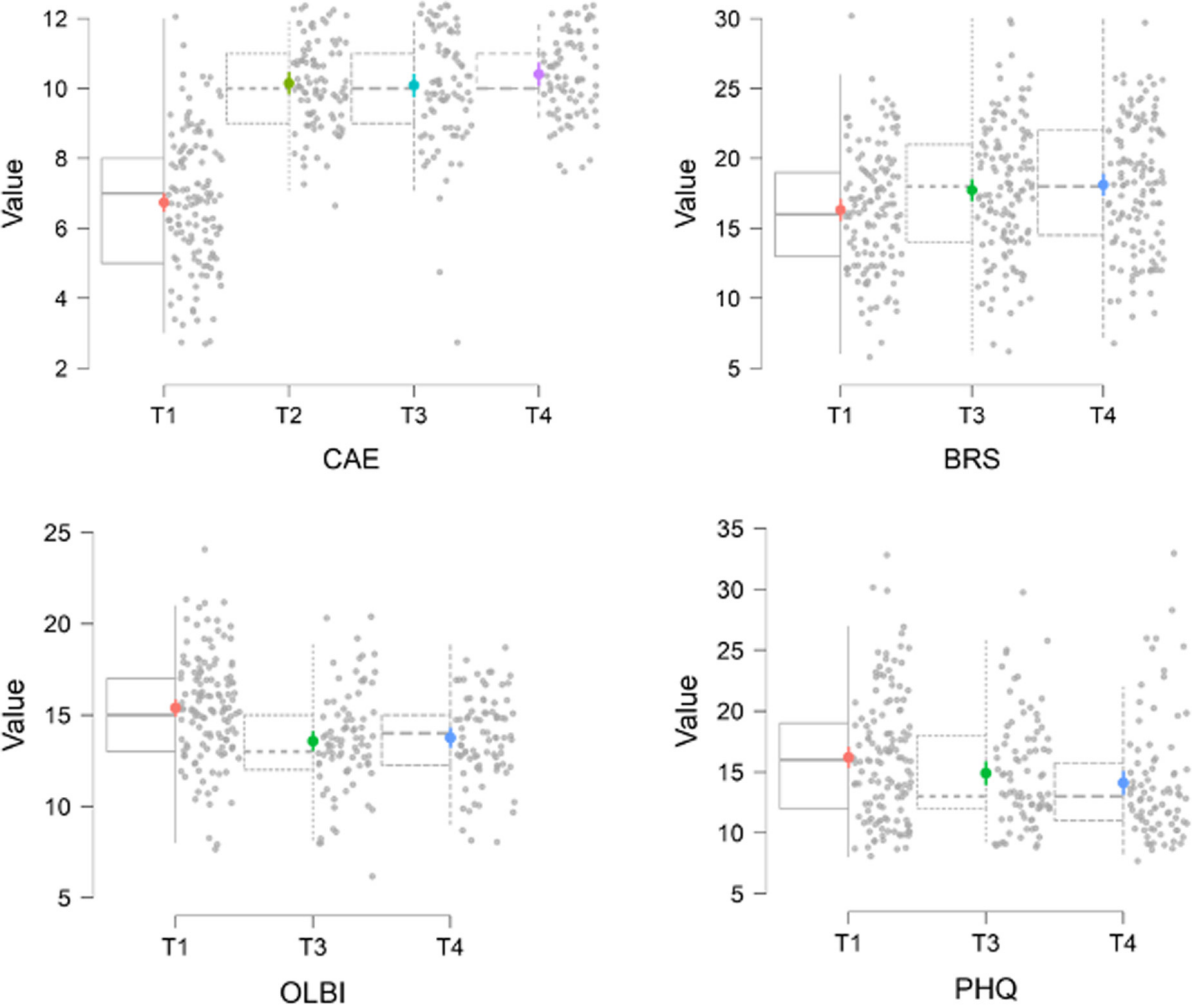
Outcome variables at each time point. CAE = Confidence in coping with adverse events; SUM_BRS = Brief Resilience Scale; SUM_OLBI = Oldenburg Burnout Inventory; PHQ_SUM = Patient Health Questionnaire (Depression).

*Secondary outcomes*. For descriptive statistics, see Table 1. For unadjusted model-fit statistics, see Table 2 and for plots, see Figure 1. All analyses indicated considerable clustering, supporting the use of random intercepts. The proportion of variance explained by all indicator variables was mixed across measures. In all cases, a large proportion of variance was explained by fixed time points explained plus random effects. Adjusting the model for control variables did not substantially alter model fit. Though association sizes were generally smaller, results in LPCF models were consistent with CC analyses. CC results are reported here. LPCF analyses and adjusted analyses are in Appendix 2.

**Table 1.** Descriptive statistics for all outcome measures.

Measure (timepoint)	Mean	Standard deviation	Min, Max
Resilience (BRS)			
T1 (n = 130)	16.33	4.34	6, 30
T3 (n = 83)	18.40	4.66	6, 30
T4 (n = 82)	19.00	4.34	12, 30
Confidence in coping with adverse wvents (CAE)			
T1 (n = 131)	6.74	1.90	3, 12
T2 (n = 93)	10.13	1.22	7, 12
T3 (n = 83)	10.06	1.66	3, 12
T4 (n = 82)	10.38	1.17	8, 12
Burnout (OLBI-abbreviated)			
T1 (n = 131)	15.40	2.87	8, 24
T3 (n = 83)	13.61	2.74	6, 20
T4 (n = 82)	13.84	2.34	8, 19
Depression (PHQ-9)			
T1 (n = 129)	7.27	5.21	0, 24
T3 (n = 83)	6.00	4.60	0, 21
T4 (n = 81)	5.32	5.02	0, 24

**Table 2.** Unadjusted complete case (CC) models: results.

Outcome	BIC	Variance (ICC)	R^2^m	R^2^c	Predictor	Contrast	beta	95% CI
Resilience (BRS)	1602	74%	7%	76%	Time	T3 versus T1	2.19***	1.51–2.87
						T4 versus T1	2.83***	2.15–3.52
Confidence (CAE)	1462	30%	52%	67%	Time	T2 versus T1	3.41***	3.05–3.77
						T3 versus T1	3.35***	2.98–3.72
						T4 versus T1	3.67***	3.29–4.04
Burnout (OLBI)	1368	62%	9%	66%	Time	T3 versus T1	−1.82***	−2.33 to −1.33
						T4 versus T1	−1.63***	−2.13 to −1.14
Depression (PHQ-9)	1685	73%	3%	73%	Time	T3 versus T1	-1.30***	-2.09 to −0.51
						T4 versus T1	-2.09***	-2.88 to −1.30

^***^ p ≤ 0.001; BIC = Bayesian Information Criterion; ICC = Intraclass Correlation Coefficient; R^2^m = R squared marginal; R^2^c = R-squared conditional; CI = confidence interval. For adjusted and LPCF analyses, see Appendix 2.

Resilience (BRS) scores increased significantly from T1 to T3 (d = 0.68) and from T1 to T4 (d = 0.89). Resilience remained static from T3 to T4, suggesting stability in initial gains (p_Holm_ = 0.13). At T1, n = 79 (60.8%) reported low resilience, n = 49 (34.6%) reported normal resilience and n = 2 (1.5%) reported high resilience. At T3, n = 27 (44.6%) reported low resilience, n = 41 (49.4%) reported normal resilience and n = 5 (6%) reported high resilience. At T4, n = 30 (36.6%) reported low resilience, n = 48 (58.5%) reported normal resilience and n = 4 (4.9%) reported high resilience.

Confidence (CAE) scores increased significantly following T1 (T2: d = 1.76; T3: d = 1.57; T4: d = 1.72). There were no further increases following T2, suggesting stability in initial gains (T2–T3 p_Holm _= 0.68; T2–T4 p_Holm _= 0.17; T3–T4 p_Holm _= 0.11).

Burnout (OLBI) scores reduced significantly from T1 to T3 (d = −0.73) and from T1 to T4 (d = −0.69). There was no difference from T3 and T4, suggesting stability in gains (p_holm_ = 0.38).

Depression scores (PHQ) indicated a significant decrease following T1 (T1–T3: d = −0.32; T1–T4: d = −0.52). There was a small, but further decrease from T3 to T4 (d = −0.29; p_holm _= 0.04). At T1, n = 37 (32.7%) participants had no depression symptoms. At T3, n = 42 (50.6%) participants had no depression symptoms and at T4, n = 46 (56.8%) had no depression symptoms. Of the 73 participants who completed all time points, at T1, n = 18 (25%) reported no depression symptoms, at T3, n = 32 (43.8%) reported no depression symptoms and at T4, n = 38 (52.1%) reported no depression symptoms.

Reactions to the workshops were overall positive (Table 3). For open-text comments provided in response to questions 5–8, see Appendix 3.

**Table 3.** Feedback following the training.

Item	Strongly disagree (%)	Disagree (%)	Neither agree/disagree (%)	Agree (%)	Strongly agree (%)	Missing (%)
1. The workshops were relevant for medical students	0 (0)	0 (0)	3 (3.2)	31 (33.3)	59 (63.4)	0 (0)
2. I learned skills in the workshops which will be useful in future	0 (0)	0 (0)	1 (1.1)	30 (32.3)	62 (66.7)	0 (0)
3. There was adequate time to cover the material	0 (0)	1 (1.1)	1 (1.1)	33 (35.5)	58 (62.4)	0 (0)
4. I found the workshops engaging	0 (0)	0 (0)	10 (10.8)	36 (38.7)	47 (50.5)	0 (0)
	Yes	No	Prefer not to say	Missing		
5. Were there any aspects of the workshops you did not find useful?	12 (12.9)	78 (83.9)	1 (1.1)	2 (2.2)		
6. Is there anything else you would have liked to see in the workshops which were not included?	19 (20.4)	72 (77.4)	1 (1.1)	1 (1.1)		
7. If you were involved in a stressful placement event, would you do anything differently as a result of attending these workshops?	85 (91.4)	4 (4.3)	0 (0)	4 (4.3)		
8. Would you recommend the workshops to other medical students	89 (95.6)	2 (2.2)	2 (2.2)	0 (0)		

## Discussion

This is the first study to evaluate a targeted intervention for medical students which is specifically tailored towards providing support with placement-related stressors, Reboot. Reboot was found to be acceptable by medical students and feasible to evaluate in this group. With high demand, recruitment targets exceeded feasibility thresholds and retention thresholds were met. Participating in Reboot was associated with significant increases in measures of resilience and confidence in coping with adverse events, and significant reductions in measures of burnout and depression. At the final time point, a greater proportion of participants were in the ‘normal’ and ‘high’ ranges for resilience, and more students were screening as asymptomatic for depression. Reactions to the workshops were generally positive, with most participants agreeing that Reboot was engaging, imparted useful skills and stating that they would do something differently, if they were involved in a stressful placement event, as a result of participating. The present findings extend existing knowledge in two main ways.

First, these positive results have relevance for the literature into supportive interventions in medical students more widely. To date, evaluations of supportive interventions for medical students have focused on generic interventions such as mindfulness and yoga.^10^ These have been criticised on conceptual grounds, suggesting they lack relevance for medical students and professionals.^11,12^ Systematic reviews have also reported mixed evidence regarding their effectiveness. For example, in a review of 39 studies testing interventions such as mindfulness, stress management training and psychoeducation in medical students, findings were mixed, with no clear evidence suggesting any individual intervention was effective for improving depression or burnout.^10^ In a systematic review of mindfulness interventions in medical students and junior doctors, no significant reductions in depression or anxiety were identified.^25^ In this context, the current study results are promising, identifying Reboot – a targeted, tailored intervention – as a potential candidate intervention suitable for investigation in further controlled studies.

Second, this is the first study to adapt and evaluate the Reboot Coaching Programme in medical students. Previous studies have evaluated Reboot in a range of healthcare student and professional groups including midwives, paramedics, obstetricians and physician associate students^15^ and critical care nurses.^18^ A workshop-only version of Reboot (without a coaching call) has also been evaluated in trainee surgeons.^16^ As with previous studies of Reboot, demand was sufficient and recruitment targets were met. Attrition was also comparable to previous studies,^18,21^ as were results regarding outcome variables.^15,16,18^ This further confirms that Reboot is a flexible intervention which can be adapted for different healthcare professional student groups with similar outcomes.

Rates of depression in our sample were higher than anticipated, with two-thirds reporting mild, moderate or severe depression symptoms at baseline. This is higher than previous studies of medical students in the UK,^26^ US,^27^ Korea^28^ and Vietnam,^29^ and could be due to two main reasons. First, while we did not recruit students with depression, students experiencing depression may have been more likely to be attracted to the study. Second, mental health in young adults has suffered over the COVID-19 pandemic period, and our sample may also reflect this wider trend.^30^

### Strengths and limitations

The study benefited from adequate statistical power and a varied participant group, who were both geographically diverse within the UK, and ethnically diverse. Rates of missing data for individual variables were also low. The study was primarily limited by a lack of a control group, which prevents conclusions regarding causality being drawn. Furthermore, we did not monitor psychoactive medications which the participants were prescribed, which may have influenced their mental health and response to the intervention. We also did not include a method of monitoring participant engagement, beyond monitoring retention rates. In future studies, researchers could consider addressing this by monitoring (1) the number of participants who have their camera on during the workshops, (2) the number of participants who speak during whole-group discussions, and the number of utterances per participant and (3) a memory test of the material covered.

### Implications for practice and research

There is a need for more effective interventions for improving mental health outcomes in medical students. The present study suggests that Reboot is a feasible intervention in this group with potential benefits for mental health outcomes in medical students. Reboot is suitable for delivery to this group in its present form and as an online intervention, could be delivered from a central hub to students from a group of medical schools simultaneously. While Reboot has only been tested in UK participants to date, it is based on CBT principles which have been found to be suitable for use in a range of countries and cultures including Asian and African nations.^31–33^ As such, it is possible that Reboot could be an internationally translatable intervention for medical students. However, further research is needed to (1) evaluate Reboot with a control group, to establish whether improvements can be attributed to the programme and (2) involve cohorts from other countries, to establish its potential generalisability.

## Conclusion

Mental health concerns affect a high proportion of medical students. Medical students are the physician workforce of the future; interventions to support, strengthen and retain this group are imperative. This is the first study to evaluate an intervention for medical students which is specifically tailored towards providing support with placement-related stressors, Reboot. Our findings indicate that Reboot is a feasible and acceptable intervention in medical students and may lead to benefits for improving resilience and confidence and reducing depression and burnout.

## Supplemental Material

sj-docx-1-scm-10.1177_00369330231213981 - Supplemental material for Reboot coaching programme: a mixed-methods evaluation assessing resilience, confidence, burnout and depression in medical students Supplemental material, sj-docx-1-scm-10.1177_00369330231213981 for Reboot coaching programme: a mixed-methods evaluation assessing resilience, confidence, burnout and depression in medical students by Judith Johnson, Lucy Pointon, Rebecca Talbot, Rebecca Coleman, Luke Budworth, Ruth Simms-Ellis, Katharina Vogt, Dialechti Tsimpida, Chandra Shekha Biyani, Reema Harrison, Gloria Cheung, Colin Melville, Vijay Jayagopal and William Lea in Scottish Medical Journal
