# Supplementary material for: Reboot coaching programme: a mixed-methods evaluation assessing resilience, confidence, burnout and depression in medical students
Source: Scott Med J. 2023 Dec 5;69(1):10–7. doi: 10.1177/00369330231213981 (PMC10986146; doi:10.1177/00369330231213981)
Supplement: sj-docx-1-scm-10.1177_00369330231213981 - Supplemental material for Reboot coaching programme: a mixed-methods evaluation assessing resilience, confidence, burnout and depression in medical students [file sj-docx-1-scm-10.1177_00369330231213981.docx]

Appendix 1: Further description of Reboot

All elements of Reboot were conducted via video platform/phone. All workshops and coaching calls were led by a Cognitive-behavioural (CBT) therapist (RC) or a Clinical Psychologist (JJ). The use of multiple, shorter intervention components which are primarily active (i.e., there is minimal didactic teaching) was designed to increase engagement and accessibility for individuals with conditions such as Attention Deficit Hyperactivity Disorder (ADHD) who may be unable to concentrate for longer periods. The online platform was also chosen to maximise accessibility for individuals with disabilities, enabling access from any location and the use of technologies to aid accessibility, such as live transcription tools.

Appendix 2: Adjusted analyses

| **Linear mixed models results (all outcomes \| unadjusted & adjusted \| complete case & last point carried forward)** | | | | | | | | | |
| --- | --- | --- | --- | --- | --- | --- | --- | --- | --- |
| **Model covariates** | **CC/LPCF** | **R^2^_M_ \| R^2^_C_** | **ICC** | **BIC** | ***n* cases \| obs.** | **Predictor** | **Contrast** | **Estimate [95% CI]** | ***p*** |
| CAE Unadjusted | CC | 52% \| 67% | 30% | 1462 | 131 \| 389 | Time | T2 vs. T1 | 3.41 [3.05-3.77] | <.001 |
|  |  |  |  |  |  |  | T3 vs. T1 | 3.35 [2.98-3.72] | <.001 |
|  |  |  |  |  |  |  | T4 vs. T1 | 3.67 [3.29-4.04] | <.001 |
|  | LPCF | 22% \| 69% | 61% | 2060 | 131 \| 524 | Time | T2 vs. T1 | 2.46 [2.14-2.77] | <.001 |
|  |  |  |  |  |  |  | T3 vs. T1 | 2.46 [2.14-2.77] | <.001 |
|  |  |  |  |  |  |  | T4 vs. T1 | 2.60 [2.29-2.92] | <.001 |
| CAE adjusted for Age \| Gender | CC | 52% \| 67% | 30% | 1474 | 130 \| 388 | Time | T2 vs. T1 | 3.39 [3.03-3.75] | <.001 |
|  |  |  |  |  |  |  | T3 vs. T1 | 3.33 [2.96-3.70] | <.001 |
|  |  |  |  |  |  |  | T4 vs. T1 | 3.65 [3.27-4.02] | <.001 |
|  |  |  |  |  |  | Age | - | 0.03 [-0.04-0.11] | 0.42 |
|  |  |  |  |  |  | Gender | M vs. F | 0.06 [-0.47-0.60] | 0.64 |
|  |  |  |  |  |  |  | O vs. F | 0.37 [-1.17-1.91 | 0.42 |
|  | LPCF | 23% \| 69% | 59% | 2054 | 130 \| 520 | Time | T2 vs. T1 | 2.48 [2.16-2.79] | <.001 |
|  |  |  |  |  |  |  | T3 vs. T1 | 2.48 [2.16-2.79] | <.001 |
|  |  |  |  |  |  |  | T4 vs. T1 | 2.62 [2.31-2.94] | <.001 |
|  |  |  |  |  |  | Age | - | 0.07 [-0.04-0.17] | 0.24 |
|  |  |  |  |  |  | Gender | M vs. F | -0.14 [-0.88-0.60] | 0.71 |
|  |  |  |  |  |  |  | O vs. F | 1.13 [-1.25-3.52] | 0.35 |
| BRS Unadjusted | CC | 7% \| 76% | 74% | 1602 | 131 \| 295 | Time | T3 vs. T1 | 2.19 [1.51-2.87] | <.001 |
|  |  |  |  |  |  |  | T4 vs. T1 | 2.83 [2.15-3.52] | <.001 |
|  | LPCF | 3% \| 82% | 82% | 2014 | 131 \| 392 | Time | T3 vs. T1 | 1.41 [0.93-1.88] | <.001 |
|  |  |  |  |  |  |  | T4 vs. T1 | 1.80 [1.32-2.27] | <.001 |
| BRS adjusted for Age \| Gender | CC | 11% \| 77% | 74% | 1603 | 130 \| 294 | Time | T3 vs. T1 | 2.23 [1.55-2.91] | <.001 |
|  |  |  |  |  |  |  | T4 vs. T1 | 2.87 [2.19-3.55] | <.001 |
|  |  |  |  |  |  | Age | - | 0.12 [-0.15-0.39] | 0.37 |
|  |  |  |  |  |  | Gender | M vs. F | 2.29 [0.44-4.13] | 0.02 |
|  |  |  |  |  |  |  | O vs. F | -1.92 [-7.61-3.78] | 0.51 |
|  | LPCF | 6% \| 83% | 82% | 2010 | 130 \| 389 | Time | T3 vs. T1 | 1.42 [0.94-1.89] | <.001 |
|  |  |  |  |  |  |  | T4 vs. T1 | 1.81 [1.34-2.29] | <.001 |
|  |  |  |  |  |  | Age | - | 0.12 [-0.15-0.40] | 0.38 |
|  |  |  |  |  |  | Gender | M vs. F | 1.98 [0.11-3.85] | 0.04 |
|  |  |  |  |  |  |  | O vs. F | -1.34 [-7.36-4.68] | 0.66 |
| OLBI Unadjusted | CC | 9% \| 66% | 62% | 1368 | 131 \| 296 | Time | T3 vs. T1 | -1.82 [-2.31 - -1.33] | <.001 |
|  |  |  |  |  |  |  | T4 vs. T1 | -1.63 [-2.13 - -1.14] | <.001 |
|  | LPCF | 3% \| 76% | 75% | 17§4 | 131 \| 393 | Time | T3 vs. T1 | -1.15 [-1.48 - -0.81] | <.001 |
|  |  |  |  |  |  |  | T4 vs. T1 | -1.04 [-1.38 - -0.70] | <.001 |
| OLBI adjusted for Age \| Gender | CC | 11% \| 66% | 62% | 1392 | 130 \| 295 | Time | T3 vs. T1 | -1.83 [-2.32 - -1.34] | <.001 |
|  |  |  |  |  |  |  | T4 vs. T1 | -1.65 [-2.14 - -1.15] | <.001 |
|  |  |  |  |  |  | Age | - | -0.12 [-0.28-0.04] | 0.13 |
|  |  |  |  |  |  | Gender | M vs. F | -0.30 [-1.40-0.81] | 0.60 |
|  |  |  |  |  |  |  | O vs. F | 1.22 [-2.13-4.56] | 0.48 |
|  | LPCF | 5% \| 77% | 76% | 1719 | 130 \| 390 | Time | T3 vs. T1 | -1.15 [-1.49 - -0.81] | <.001 |
|  |  |  |  |  |  |  | T4 vs. T1 | -1.05 [-1.39 - -0.71] | <.001 |
|  |  |  |  |  |  | Age | - | -0.12 [-0.28-0.05] | 0.17 |
|  |  |  |  |  |  | Gender | M vs. F | -0.12 [-1.26-1.01] | 0.83 |
|  |  |  |  |  |  |  | O vs. F | 0.84 [-2.82-4.50] | 0.66 |
| PHQ-9 Unadjusted | CC | 3% \| 73% | 72% | 1685 | 131 \| 296 | Time | T3 vs. T1 | -1.30 [-2.09 - -0.51] | .001 |
|  |  |  |  |  |  |  | T4 vs. T1 | -2.09 [-2.88 - -1.30] | <.001 |
|  | LPCF | 1% \| 82% | 82% | 2098 | 131 \| 393 | Time | T3 vs. T1 | -0.80 [-1.32 - -0.28] | .003 |
|  |  |  |  |  |  |  | T4 vs. T1 | -1.32 [-1.84 - -0.80] | <.001 |
| PHQ-9 adjusted for Age \| Gender | CC | 7% \| 73% | 71% | 1687 | 130 \| 295 | Time | T3 vs. T1 | -1.32 [-2.11 - -0.53] | .001 |
|  |  |  |  |  |  |  | T4 vs. T1 | -2.11 [-2.90 - -1.32] | <.001 |
|  |  |  |  |  |  | Age | - | 0.06 [-0.24-0.36] | 0.68 |
|  |  |  |  |  |  | Gender | M vs. F | -0.10 [-2.16-1.97] | 0.93 |
|  |  |  |  |  |  |  | O vs. F | 7.55 [1.20 – 13.89] | 0.02 |
|  | LPCF | 4% \| 82% | 82% | 2093 | 130 \| 390 | Time | T3 vs. T1 | 0.81 [-1.33 - -0.28] | .003 |
|  |  |  |  |  |  |  | T4 vs. T1 | -1.33 [-1.86 - -0.80] | <.001 |
|  |  |  |  |  |  | Age | - | 0.09 [-0.21-0.40] | 0.56 |
|  |  |  |  |  |  | Gender | M vs. F | 0.09 [-1.98-2.15] | 0.94 |
|  |  |  |  |  |  |  | O vs. F | 7.23 [0.57-13.88] | 0.04 |
|  |  |  |  |  |  | Prev. Mental Health Support | Y vs. N | 4.53 [2.81-6.25] | <.001 |
| CC = complete cases; LPCF = last point carried forward; ICC = intraclass correlation coefficient; R^2^_M_ \| R^2^_C_ = R^2^_C_ marginal/conditional; 95%CI = 95% confidence interval. *CAE = Confidence in coping with adverse events questionnaire. BRS = Brief Resilience Scale. OLBI = Oldenburg Burnout Inventory (brief version). PHQ-9 = Patient Health Questionnaire (Depression).* | | | | | | | | | |

Appendix 3: Open text comments

All comments from the 12 (12.9%) people responding YES to the question: ‘Were there any aspects of the workshops you did not find useful?’ (sic)

- I feel the sessions were too long for the numbers of activities? I found i was sitting alot as there was too much time for each acitivyt - so maybe more activities?
- problem solving exercise
- I don't think the example of needlestick injury for problem solving was too good because there is a set protocol so it was difficult to discuss different ideas as we all had the same idea
- Think it would be better if everyoone had to have their cameras on although I appreciate that it's easier said than done
- CBT theory
- The situation given for activity 9 doesn't make the best use of the tool/flowchart, because with needle stick injuries there's really only 1 way to deal with it. The flow chart/tool is great but would give more useful discussion with a different example scenario.
- Some of the activities felt unnecessary e.g. pie chart
- Basic science, in depth explanations would’ve been more interesting to me personally
- Going over some medical basics such as flight or fight
- I found that the examples were focused on coping with medical errors, which isn't really a source of anxiety or that applicable to me as a medical student
- Resilience strategies i.e. practicing gratitude

All comments from the 19 (20.4%) people responding YES to the question ‘Is there anything else you would have liked to see in the workshops which were not included?’ (sic)

- A sheet at the back listing the methods we talked through, breathing etc
- More topics related to dealing with anxiety for the future rather than dealing with stressful events.
- Maybe more opportunities to discuss our own personal experiences and how we could have applied the strategies to these
- Ways of dealing with grief and specific adverse events in personal life
- Being able to approach and deconstruct stressful events we'd previously had
- Tips for managing sadness and processing sad events, which are different to stressful events - for example, I get overly sad/upset when I hear of sad situations, especially when they don't actually effect me.
- How to implement toolkit for neurodivergent folk.
- potentially more about supporting colleagues as well, although understand this may be beyond the scope of the programme
- More on chronic burnout
- It would be interesting to know more about the research into traumatic/adverse events in doctors and how these impact the workforce. I'd also love to learn more about time frames and in general how long it can take to recover from stressful events.
- Further exploration of how to stop/change negative habits you already have in place. For example I have trouble inserting cannulas after a bad experience, how to I deal with that and move on?
- The same lessons and activities but with mention and discussion about how these strategies work alongside concurrent mental health conditons that students may have (adhd) or be vulnerable to (anxiety disorders)
- Some more practical exercises
- How to deal with anger about a situation. It's difficult to implement some of the techniques when you are angry
- coping with issues whilst diagnosis of ADHD, Depression and anxiety. As it can be difficult to even see situations as potentially harmful
- If the workbook was a word document to allow for easier editing
- CBT styled teaching and more strategies that can be incorporated in solving negativity
- Perhaps, more practical examples of using stress relievers.

All comments from the 85 (91.4%) people responding YES to the question ‘If you were involved in a stressful placement event, would you do anything differently as a result of attending these workshops?’ (sic)

- yes i would try the responsibilty pie chart to try and understand why the stressful event occured and actively recognise my negative thinking patterns so that i can implement the correct self help methods. Ill difinatelty be more kinder to myself as a result of the workshop and will be revisiting the content when i find myself unsure of how i can help myself
- practise putting things into perspective and also might use the responsibility pie chart
- have tools to think about it and be able to deal with the impacts
- Distract myself then go home and write down good things that happened today
- implement mood boosting technologies, recognise negative thinking patterns, look for help on the website links recommended
- use the pie chart
- I know where to go for help
- Not place so much blame on myself. Take a step back. Talk to family and friends.
- I would take some time to address it with myself and maybe a colleague
- I would focus on my breathing, remove myself from the enviroment and seek closeness
- reflect on the event in a better way
- Use the counteractive thoughts to assist in destressing
- I think the responsibility pie chart would have really helped me as I was blamed for something that was not my fault for weeks and it was difficult to remember that actually I only had a small part
- I would know where to look for support and how to manage myself
- I feel I have more understanding of the strategies and how to use them
- Try some new strategies we discussed
- Take some time to think through the situation as a whole
- Using the breathing method and pie chart
- Not ruminate and instead have better coping strategies
- some of the self-esteem boosting techniques and positive mind strategies
- Use the responsibility pie chart to fairly allocate responsibility for the event, use mood boosting activities, and use strategies for managing negative thinking habits
- Better techniques to help get over the anxiety
- Now have better coping mechanisms and access to support
- jot down thoughts and emotions at the time so i can couteract them later, jot down who else assigned responsibility, initiate toolkit for
- knowinf where to go for support
- Use the pie chart to balance my feelings of guilt vs my feelings of 'this isn't fair'
- I will try to manage my stress looking both at short term and long term strategies
- Taking a moment for breathing exercises, writing down contributing factors after a mistake
- Not take such a large proportion of the blame
- Use CBT approaches to deal with the event at the time and afterwards long term
- Utilisation of the toolkit to manage short and long-term stress will be very useful.
- I would start write in my gratitude journel to be grateful for what i have
- I feel I would now know how to avoid taking full responsibility for the event. I believe I now have the tools to manage anxious thoughts that may present as a result of a stressful event.
- I have more strategies to implement and have a greater awareness of the negative thinking patterns I tend to go down.
- Rationalise blame and realise that I am not 100% to blame in most situations
- Reframing negative thinking patterns, focusing on the three categories of coping strategies discussed
- I would use the breathing exercises to clam myself and write out the pie chart to be more aware of all the various causes of the event. I think it was also helped me identify negative thinking habits in my daily life better too. I also like the concept of setting a timer for 5 minutes to motivate a positive thinking habit; I plan to set a 5min timer to tidy my room and motivate me out of a negative thinking habit.
- Construct a strategy of how I was going to deal with the repercussions rather than just winging it (even if both involved the same exercises e.g. sport, distraction, friends/family)
- Make sure to speak to a colleague and avoid negative thinking habits
- Feel more equipped to cope with event and rationalise my emotional response
- I have tools to gain emotional support and recovery
- think about thoughts and emotions, have an immediate plan to deal with emotions and behaviours and a longer term plan
- I will use the tools to promote resilience and positive thinking
- Stop ruminating on the event and use the strategies learnt to reflect and move forward
- Self statements to counteract negative thoughts and responsibility pie chart.
- Check out Second Victim support - I wasn’t aware this resource was available and that video really helped to understand that mistakes do happen and they aren’t just one persons responsibility.
- Make use of immediate and long term strategies
- Less likely to think I had absolute responsibility
- I would be able to think about it more clearly and call upon the toolkit outlined in the workshops to apply strategies to help me better deal with the event
- I would work more on trying not to put all the blame on myself and I would work through my strategies of boosting my mood
- I would employ some of the coping strategies mentioned, take time to breath and get away from the environment and ensure i refelct on the event afterwards so I properly process it
- Try and do positive things to boost my mood, and avoid rumination by practicing the activities we talked about in the workshop
- Better ways of coping with it and now know who/ what to consult if in that situation
- Not blame myself. There are other contributing factors, I was not the only source of blame.
- more positive thinking and reflection
- Better coping strategies
- I would use the techniques we have practiced through the exercises in order to process what had happened in a more balanced, constructive way. As a result, I would probably respond more pragmatically to this situation if it occurred.
- Divide responsibility
- I would communicate better and take a break for a few minutes to gather my thoughts
- use the second victim site and find comfort within the many strategies we went through
- implement some of the techniques learnt
- I would try to place it in perspective by trying to see the positives. I would also recognise that ruminating may not be helping
- Know who to talk to and where to seek advice
- The idea of giving myself time to worry, trying to retrain the way i think about things and having a preset plan to cope with these experiences is comforting and i look forward to using them.
- Better stress management
- I would feel more able to assign responsibility more objectively instead of just blaming myself. I would also be able to recognise if I was ruminating, or generalising and I would be able to practice things should as positive statements to help with these thoughts.
- second victim support
- I would initially take time to breath and reset my mind, then ensure that I debrief with a colleague
- After this workshop, I would definitely reframe the event differently in my head, in a more balanced and constructive way. I would also engage in healthier methods of coping like the ones we discussed in the workshop.
- Would be able to employ techniques and speak from my learning
- strategies to combat negative thinking habits and behaviours
- Especially the ascribing responsibility exercise. Trying to notice patterns of negative thinking
- Know how to cope
- Responsibility pie chart, reflect, avoid harmful thought patterns
- Think of some immediate mood boosting activities to ease the stress
- remind myself about my self esteem pillars
- I would better be able to explain the situation and manage my emotions afterwards
- Use some of the strategies and seek help
- Seek out my supervisor earlier. Work on my mindset, thoughts and destressing afterwards. Making a plan of how to overcome fears/unhelpful avoidance strategies that may be related to the event.
- Ordering who is to blame with myself last rather than putting myself first to blame
- pie chart of responsibility
- positivity and breathing
- I think I’d try to deal with the stress before the problem

All comments in response to a final item, ‘Please add any additional comments related to this workshop here: the question’

- i enjoyed the workshop as they provided a safe space to discuss some of the difficult issues we face as med student that were expected to just move on from. I found the exercises in the workbook relevant to situtions id face on placement so it was nice to reflect on how i would react which is usually more negative and self critical and instead how i could react in more postivitve way.
- Great workshop, I feel something like this should be compulsory for all medical students as it provides strategies that help deal with difficult situations that are inevitable in the medical field. I have heard of countless doctor that have a mentally difficult time as a doctor hence training like this should be more common.
- I don't think its good that our full names were displayed during the zoom call as i feel this compromises confidentiality
- n/a
- love the relaxed and comfortable environment of the zoom calls
- The sessions were quite long: 1.5 hours would be better.
- Thank you so much for hosting these sessions!
- Great to go over all the exercises - would recommend to all other medical students
- I think the sessions would be quicker if we were encouraged to completed the exercises before the session and maybe make them an hour long with more breakout rooms/discussion.
- The sessions were quite long and I found it hard to focus for the full 2 hours with only a 5 min break after a full day of placement!!!
- Engaging tutor and good balance of theory and interactive exercises
- N/A
- really helpful workshop that all medical students should try
- I liked the scenarios as they were quite realistic. But it was hard to implement the tasks in daily life as we are all on summer holidays as students and don't face potentially adverse scenarios.
- Thanks!
- so so useful thank you so much!! really helped to organise thoughts and create a systematic way of managing stressful events. scenarios and case examples were particularly useful as they felt really relatable and realistic things that can happen in our future practice
- Some really practical tips which I will definitely use. Glad I took part. Thank you!
- Thank you!
- Overall, the workshops were fantastic and very high quality. I learned a lot about resilience and identifying negative thought habits and I am already seeing the benefit of this to my mental wellbeing.
- Some pre reading before the second workshop to give a little bit of information about the ideas explored may help to solidify the learning from the second workshop
- Really helpful in the short amount of time we had. Rebecca was so helpful and made sure to point out resources that are helpful even no matter what medical school we attend
- Very useful, everyone needs this before starting placements!
- Thank you!
- Thank you for your time
- The workshop emphasised the importance of mindfulness and mental health. Knowing the scientific basis behind each tool that was taught to combat stress has reinforced the importance of resilience and has further motivated me to practice mindfulness on a daily basis.
- This has been very useful.
- It was really helpful thank you. It would be great if we could get a copy of the teaching slides too? As a reminder of elements of the toolkit e.g. pillars of self esteem to refer back to in the future.
- Great information and solution for mood management in stressful work life
- Would be beneficial to have more of the info from the slides in the workbook, especially for the second session
- Amazing workshop! Found it very helpful and comforted me to know there are people with similar problems to me and an initiate to help with this (I have to admit my medical school gives us next to no training/advice on these sorts of situations)
- It was really useful and informative, thank you!
- I frequently found myself zoning out, this may just be due to my tiredness. However, I think more energetic delivery may have helped.
- I found these workshops incredibly useful and beneficial. I think the strategies and learning involved can be carried forward on a very practical level with regards to changing our mindsets, building our resilience and contributing to maintaining wellbeing.
- We ran out of time both workshops- timings may be an issue
- I wish this workshop was part of the medical curriculum, these lessons have so much potential to protect students from feeling alienated and powerless in their med school journey and beyond
- I found the workshop really helpful. It was really validating and useful to know that there are coping strategies out there that can help. It was really helpful to have something made specifically for medical students as I think we are faced with a lot of anxiety inducing situations and before this I haven't really had any support to equip myself for these situations in this much depth. The facilitator was really friendly, approachable and understood what is useful for medical students.
- 2 hours felt slightly too long to be able to concentrate and sometimes felt a little drawn out, so I would have found it more engaging to have only been 1.5 hours or so and a little more fast paced if possible
- I really enjoyed the workshops - I found it useful, engaging, and thorough. My only feedback would be that 2 hours felt quite long to be concentrating; I would have definitely preferred 4 sessions of 1 hour each in order to better engage and then reflect on the information I learnt, so that it was not too much in one go. I definitely feel this workshop's use case could also be expanded from just adverse events medical students face as sometimes it was difficult to relate to many of the examples as we just don't encounter adverse events that often in comparison to other more general stressful events.
- Really enjoyed! Just found session 2 to contain a lot of information and was dense so hard to stay focussed and follow through
- Thank you- really nice workshops
- I would struggle to remember the techniques in the moment
- Thank you so much for this session, it was beyond useful! I can already see it benefitting me personally and at work
- Thank you
- N/A
- tutor was really lovely
